# Factors Affecting the Awareness of Diabetic Retinopathy: An Observational Cross-Sectional Study

**DOI:** 10.7759/cureus.59020

**Published:** 2024-04-25

**Authors:** Rihab A Ghanma, Rania Al-Asa'd, Abeer Mohammad, Mohannad Al Qararah, Abdullah Bani Issa

**Affiliations:** 1 Ophthalmology, Jordanian Royal Medical Services, Amman, JOR; 2 Diabetes and Endocrinology, Jordanian Royal Medical Services, Amman, JOR

**Keywords:** optometrist, screening, nutritionist, hba1c, diabetes mellitus, awareness

## Abstract

Background: Diabetes mellitus (DM) is a chronic metabolic disease characterized by elevated blood glucose levels. Over time, it can lead to serious complications in the eyes, heart, blood vessels, kidneys, and nerves, being the leading cause of blindness among working-age patients.

Aim and methods: This descriptive observational cross-sectional study aims to evaluate the factors affecting the awareness of the general adult diabetic population about their chronic disease and its complications. A survey was distributed anonymously among diabetic patients in different parts of Jordan over four months (January 2023-April 2023), targeting diabetic patients (DMT2 or adults DMT1). The link was sent via WhatsApp to willing candidates. Data collected included age, sex, region, education, home blood sugar (BS) testing, knowledge about cumulative blood sugar test (HbA1c), eye affection by DM, optician role, and doctor and nutritionist follow-up visits. A chi-squared test or Fisher’s exact test explored the association between categorical data; a z-test was applied for column proportion differences. An alpha level of 0.05 was deemed statistically significant. IBM SPSS Statistics for Windows, Version 28 (Released 2021; IBM Corp., Armonk, New York) was used for data analysis.

Results: The sample comprised 447 diabetic adults aged 18-80 years. The majority were school-educated or school leavers (278; 62.2%); 20 (4.5%) held a master’s or PhD degree. The largest group had DM for one to five years. Insulin was the sole treatment for 188 patients (42.1%), while oral hypoglycemic agents (OHA) were used by 170 patients (38%) as the only anti-DM medication. A total of 174 patients (38.9%) had never been seen by an ophthalmologist, and 153 (34.2%) believed an optometrist checkup suffices. Although 381 (85.2%) reported knowing DM affects the eyes, 272 (60.9%) believed they needed to see an ophthalmologist only when experiencing eye symptoms. Less than half (186; 41.6%) had an HbA1c reading of 7% or less. There was a significant correlation between education level and awareness of DM and diabetic retinopathy (DR): HbA1c, regular home BS checkups, early DR symptoms, and optometrist visits. Significant variations in awareness were noted across Jordan's major areas. Diabetics with abnormal HbA1c who visited a nutritionist were almost triple those who did not. The main information source about DM and DR was the treating physician for 298 (66.7%) respondents.

Conclusion: Awareness of DM and DR in Jordan is not satisfactory for assisting patients in their long journey with minimal complications. A national awareness campaign utilizing social media and a sustainable screening program prioritizing the north, south, and middle regions of Jordan are needed.

## Introduction

Diabetes mellitus (DM) is a chronic, metabolic disease characterized by elevated levels of blood glucose. Over time, it can lead to serious complications affecting the eyes, heart, blood vessels, kidneys, and nerves. The most common form is type 2 diabetes (T2DM), characterized by insufficient insulin production or insulin resistance in the body. The number of cases and the prevalence of diabetes have been steadily increasing over the past few decades [1]. DM is associated with a twofold excess risk of vascular disease [2]. Diabetic retinopathy (DR) is a microvascular complication of diabetes and the leading cause of blindness among working-age patients, with a worldwide prevalence of 34.6% among diabetics [3]. By 2040, of the 600 million people worldwide with DM, 400-500 million will live in low- and middle-income countries [4]. Population surveys consistently show that many diabetics are unaware of their risk for DR and other complications [4].

## Materials and methods

A questionnaire, prepared by ophthalmologists and endocrinologists, was validated by experts before distribution. The link was sent to colleagues, including doctors, nurses, quality officers, and public health workers in the Royal Medical Services (RMS), who volunteered to distribute the link. Patients visiting the emergency room and general practitioner's outpatient clinics were approached, and the survey was explained to those with DM. They were asked for permission to send the link to their phones. Responses were collected over a period of 4 months (January 2023-April 2023), targeting diabetic patients (DMT2 or adults DMT1). The link was sent via WhatsApp to willing candidates who shared their numbers. Diabetics without access to WhatsApp, especially elderly individuals in rural villages, were reached via their accompanying family members. The questionnaire stated it was an anonymous survey, described the goals, and mentioned the health workers’ institute involved. Data collected included age, sex, region, education, home blood sugar (BS) testing, knowledge about the cumulative blood sugar test (HbA1c), eye affection by DM, optician role, and doctor and nutritionist follow-up visits. Categorical data were expressed in frequency and percentages. A chi-squared test or Fisher’s exact test was used to explore associations between categorical data, in addition to a z-test for column proportions differences. The alpha level was set at 0.05 for statistical significance, and SPSS software v. 28 was used for data analysis. Finally, respondents were asked to identify their main source of information about DM and DR.

The sample size was calculated (www.qualtrics.com) with a confidence level of 95%, a margin of error of 5%, and a population size of 760,000. Distribution was stopped once the ideal calculated sample size was exceeded.

Ethical approval for this study was obtained from the Human Research Ethics Committee at the Royal Medical Services, meeting number 1/2023.

## Results

**Figure 1:**
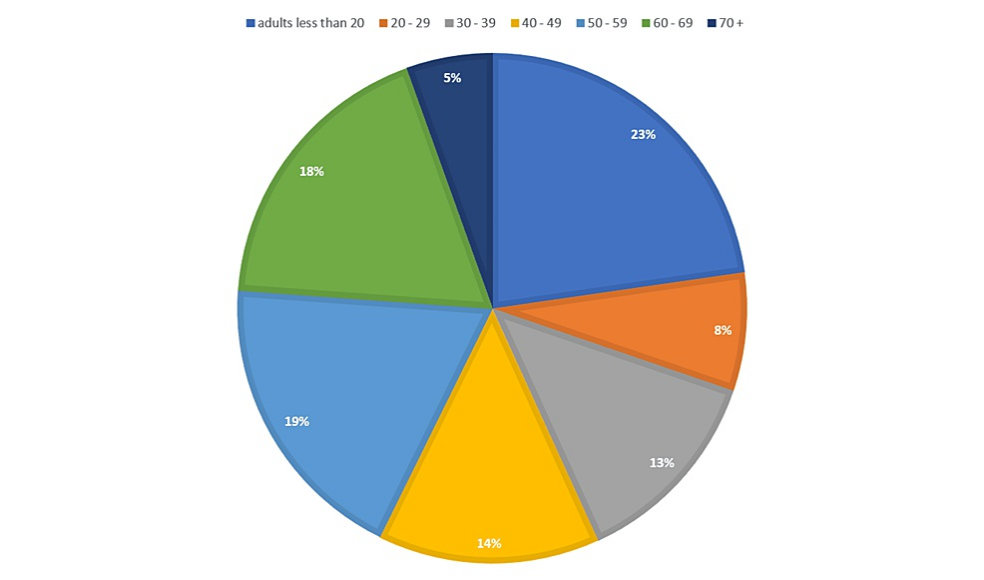
The age distribution of population in years (percentage out of the total population group)

**Figure 2:**
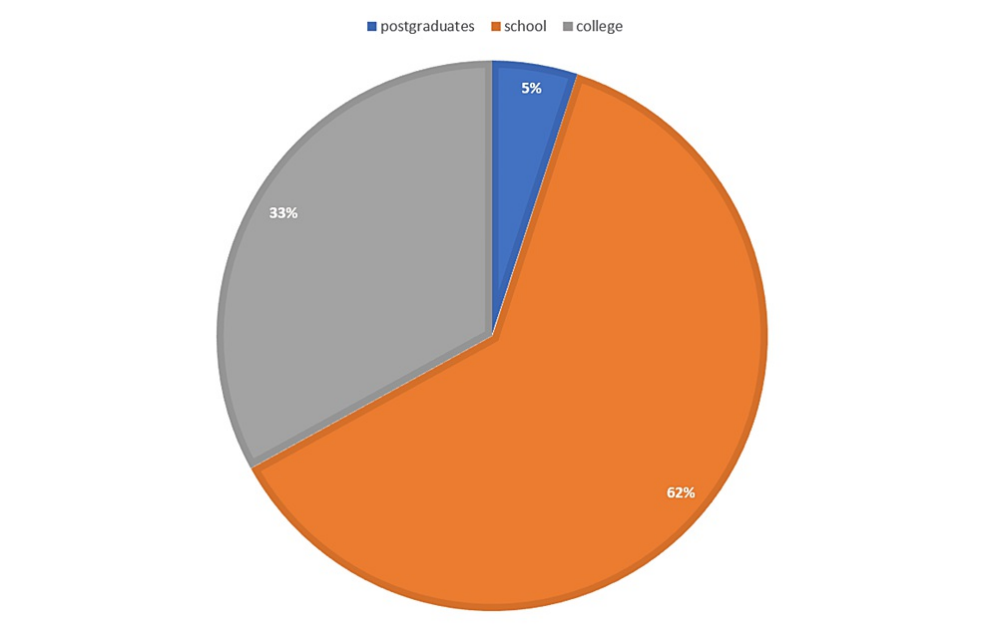
Distribution of levels of education of the study group (percentage of school-educated or less, college, and post-graduates in the population group)

The sample consisted of a total of 447 diabetic adults aged 18-80 years, as shown in Figure 1. Of the respondents, 236 (52.8%) were females. The level of education of the participants is illustrated in Figure 2. The majority, 278 (62.2%), were school-educated or school leavers, while 20 (4.5%) respondents had received a master’s or PhD degree.

**Figure 3:**
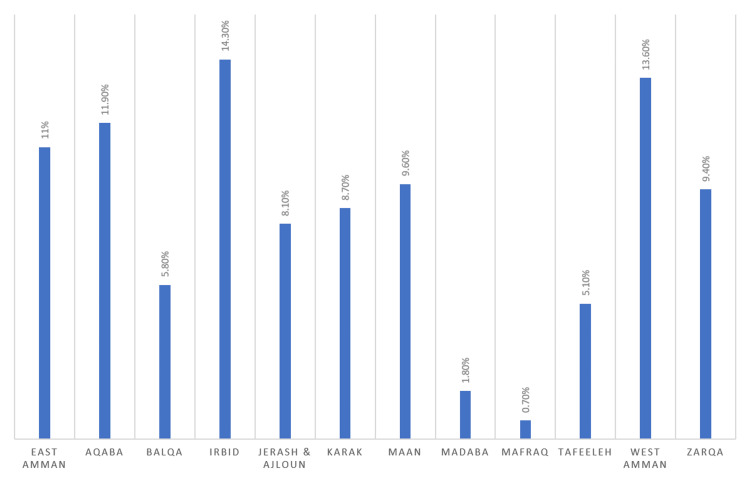
Distribution of the group of patients according to major areas in Jordan (in percentage).

The study group was selected from all major regions of the Hashemite Kingdom of Jordan. However, the groups were not equal, mainly due to differences in population density in various regions. The distribution in percentage across the major areas is illustrated in Figure 3.

The duration of DM in the group was variable. The largest group had been diagnosed with DM for between one and five years. The smallest group, forming only 14.1% of the respondents (63 patients), were those recently diagnosed (less than a year). The remaining groups shared almost equal portions, with 24.6% (110 patients) having DM between five and ten years, and 24.4% (109 patients) diagnosed with DM for more than 10 years. Insulin was the only treatment for 188 (42.1%) patients, whereas OHAs were used by 170 (38%) as their only anti-DM medication. Less than a fifth, 70 patients (15.7%), used both insulin and OHA. Seventeen patients followed a low-glucose diet only and two used herbal medicine. More than half, 236 (52.8%) of the patients have at least one immediate family member diagnosed with DM.

When asked if they had a regular appointment with the treating DM doctor, more than a third, 165 (36.9%) stated that they were not connected to a doctor and had no appointment. Two-thirds (298 patients) had appointments that varied in frequency from every three months to a year. In all, 174 (38.9%) patients had never been seen by an ophthalmologist; the rest had already been seen in an eye clinic. Furthermore, 68 (34.2%) of respondents believe that an optometrist checkup is enough and substitutes for an eye doctor check.

Although 381 (85.2%) reported that they know DM affects the eyes, 272 (60.9%) believed they needed to see the ophthalmologist only when they had eye symptoms. Less than a third, 126 (28.2%), knew that they needed to have their eyes checked immediately after DM diagnosis. Only eight patients thought that the eyes did not need to be checked before 10 years of DM diagnosis.

**Figure 4:**
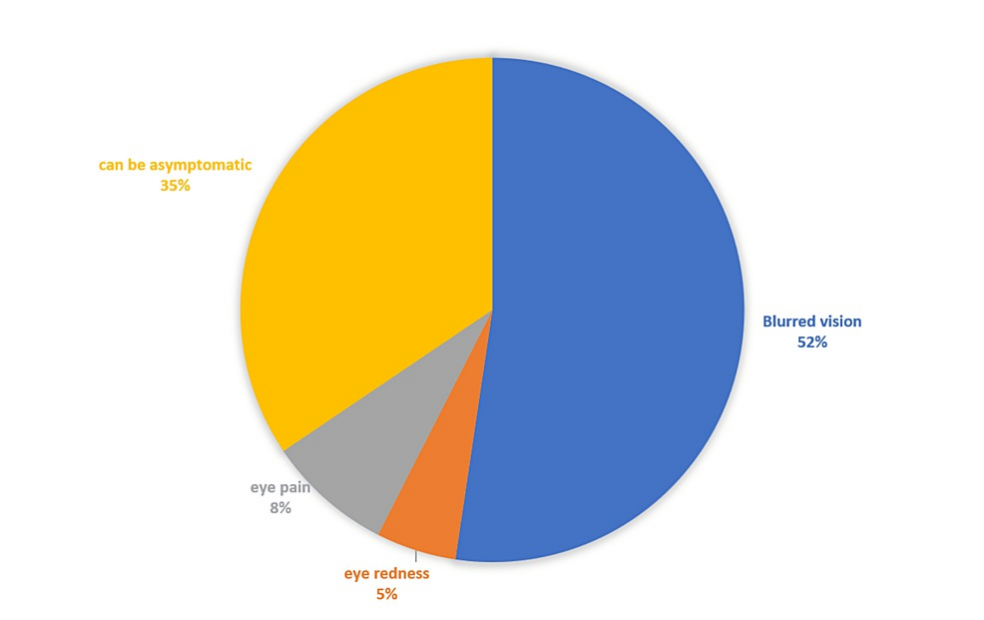
The study group's response to a question about the early effects of DM on the eyes (in percentage).

Regular home blood sugar checkups were available and followed by more than two-thirds, 304 (68%) of respondents. However, 114 (25.5%) had no idea whether DM had caused them any complications, whereas 215 (48.1%) thought they have no complications from DM. Early effects of DM on the eyes were reported by the respondents as shown in Figure 4.

The cumulative blood sugar level (HbA1c) was well known to most candidates: 440 (98.4%) understood what it means, and most knew their readings. However, less than half of the respondents, 186 (41.6%), had an HbA1c reading of 7% or less. On the other hand, 188 (42%) of the respondents knew that their readings were 8+ (86 (19.2%)) or 9+ (153 (34.2%)).

Only 82 (18.4%) responded “yes” to the general question of whether DM could have already affected the eyes before diagnosis, while almost half of the responses, 218 (48.7%), were “I have no idea.” Furthermore, 297 (66.4%) knew that DR is related to DM control, whereas almost a third, 131 (29.3%) answered “I don’t know”, and 19 (4.3%) thought they are not related. However, almost two-thirds, 281 (62.9%), had already visited a DM educator nurse at least once since their DM diagnosis.

Statistically, there was a significant correlation between the level of education and awareness about DM and DR, including HbA1c, regular checkup of BS at home, early symptoms of DR, and optician visits. However, there was no significant difference between all three levels of education concerning the awareness of DR that could already be present upon DM diagnosis, as 60% of highly educated patients admitted that they lacked information concerning this fact (Table 1).

**Table 1:** Analysis of responses according to the educational level. N: number of patients, DM: diabetes mellitus, DR: diabetic retinopathy, BS: blood sugar *p<0.05 ** p<0.001

Variables	Categories	Educational level			Test value	p-value
		Secondary and less, N(%)	Bachelor or diploma, N(%)	Higher degrees, N(%)		
Would an optician visit instead of an eye doctor be enough to detect DR?	No	162(58.7)	112(76.2)	16(80.0)	14.942	0.001
	Yes	114(41.3)	35(23.8)	4(20.0)		
Your visit to DM treating doctor	Every 3 months	110(39.9)	41(27.9)	8(40)	13.536	0.028
	Every 6 months	10(3.6)	2(1.4)	0(0.0)		
	Annually	54(19.6)	50(34.0)	5(25.0)		
	No specific time	102(37.0)	54(36.7)	7(35.0)		
Do you regularly check your BS at home?	No	72(26.1)	66(44.9)	4(20.0)	16.981	<0.001
	Yes	204(73.9)	81(55.1)	16(80.0)		
Is DR related to the control of BS?	Maybe no	95(34.4)	31(21.1)	4(20.0)	12.108	0.017
	No	9(3.3)	10(6.8)	0(0.0)		
	Yes	172(62.3)	106(72.1)	16(80.0)		
Would it be possible to have DR already started once the diagnosis of DM is established?	No	88(31.9)	53(36.3)	4(20.0)	4.842	0.304
	Yes	46(16.7)	30(21.2)	4(20.0)		
	I don't know	142(51.4)	62(42.5)	12(60.0)		
Have you ever heard about HbA1c? If yes, what is your latest reading?	Never informed	8(2.9)	4(2.7)	1(5.0)	95.263	<0.001
	I don’t know	8(2.9)	1(0.7)	0(0.0)		
	<5%	6(2.2)	9(6.1)	5(25.0)		
	About 6%	33(12.0)	23(15.6)	7(35.0)		
	About 7%	37(13.4)	59(40.1)	4(20.0)		
	About 8%	56(20.3)	26(17.7)	3(15.0)		
	>9%	128(46.4)	25(17.0)	0(0.0)		
Early DR presents with	Redness	12(4.3)	10(6.8)	1(5.0)	13.094	0.042
	Pain	28(10.1)	8(5.4)	0(0.0)		
	Blurry vision	145(52.5)	80(54.4)	6(30.0)		
	Asymptomatic	91(33.0)	49(33.3)	13(65.0)		

For comparison of the country’s regions, Jordan was divided into three major areas: north, middle, and south. A significant variation in awareness was noticed between these regions in the responses to all the questions, except for the regular BS home checkups. Table 2 presents these data in detail.

**Table 2:** Analysis of responses according to the region of the country. N: number of patients, DM: diabetes mellitus, DR: diabetic retinopathy, BS: blood sugar *p<0.05 ** p<0.001

Variables	Categories	Regions			Test value	p-value
		Middle, N(%)	South, N(%)	North, N(%)		
Would an optician visit instead of an eye doctor be enough to detect DR?	No	123(66.1)	118(76.6)	49(47.6)	23.101	<0.001
	Yes	63(33.9)	36(23.4)	54(52.4)		
Your visit to DM treating doctor	Every 3 months	82(44.1)	57(37.0)	20(19.4)	54.066	<0.001
	Every 6 months	5(2.7)	6(3.9)	1(1.0)		
	Annually	46(24.7)	16(10.4)	47(45.6)		
	No specific time	53(28.5)	75(48.7)	35(34.0)		
Do you regularly check your BS at home?	No	55(29.6)	52(33.8)	35(34.0)	0.910	0.635
	Yes	131(70.4)	102(66.2)	68(66.0)		
	Maybe no	47(25.3)	42(27.3)	41(39.8)	25.561	<0.001
Is DR related to the control of BS?	No	7(3.8)	1(0.6)	11(10.7)		
	Yes	132(71.0)	111(72.1)	51(49.5)		
Would it be possible to have DR already started once the diagnosis of DM is established?	No	68(36.8)	60(39.0)	17(16.5)	23.357	<0.001
	Yes	38(20.5)	28(18.2)	15(14.6)		
	I don't know	79(42.7)	66(42.9)	71(68.9)		
Have you ever heard about HbA1c? If yes, what is your latest reading?	Never informed	6(3.2)	5(3.2)	2(1.9)	28.817	0.004
	I don’t know	2(1.1)	4(2.6)	3(2.9)		
	<5%	11(5.9)	6(3.9)	3(2.9)		
	About 6%	37(19.9)	16(10.4)	10(9.7)		
	About 7%	49(26.3)	35(22.7)	16(15.5)		
	About 8%	38(20.4)	29(18.8)	18(17.5)		
	>9%	43(23.1)	59(38.3)	51(49.5)		
Early DR presents with	Redness	7(3.8)	7(4.5)	9(8.7)	19.235	0.004
	Pain	18(9.7)	9(5.8)	9(8.7)		
	Blurry vision	99(53.2)	95(61.7)	37(35.9)		
	Asymptomatic	62(33.3)	43(27.9)	48(46.6)		

Significant variations in nutritionist visits were reflected in the respondents' answers on three key points: visiting the diabetic doctor, taking regular home readings of BS, and awareness of HbA1c (Table 3). Notably, diabetics with abnormal HbA1c who visited the nutritionist (176 patients) were almost triple those who did not see one (62 patients).

**Table 3:** Analysis of responses in relation to nutritionist/educator visits effect. N: number of patients, DM: diabetes mellitus, DR: diabetic retinopathy, BS: blood sugar *p<0.05 ** p<0.001

Variables	Categories	Nutritionist/educator visit		Test value	p-value
		No, N(%)	Yes, N(%)		
Would an optician visit instead of an eye doctor be enough to detect DR?	No	117(70.9)	173(62.2)	3.450	0.063
	Yes	48(29.1)	105(37.8)		
Your visit to DM treating doctor	Every 3 months	52(31.5)	107(38.5)	11.403	0.010
	Every 6 months	3(1.8)	9(3.2)		
	Annually	33(20.0)	76(27.3)		
	No specific time	77(46.7)	86(30.9)		
Do you regularly check your BS at home?	No	69(41.8)	73(26.3)	11.059	0.001
	Yes	96(58.2)	205(73.7)		
Is DR related to the control of BS?	Maybe no	51(30.9)	79(28.4)	0.513	0.774
	No	6(3.6)	13 (4.7)		
	Yes	108(65.5)	186(66.9)		
Would it be possible to have DR already started once the diagnosis of DM is established?	No	52(31.7)	93(33.5)	0.164	0.921
	Yes	30(18.3)	51(18.3)		
	I don't know	82(50.0)	134(48.2)		
Have you ever heard about HbA1c? If yes, what is your latest reading?	Never informed	7(4.2)	6(2.2)	42.917	0.001
	I don’t know	7(4.2)	2(0.7)		
	<5%	16(9.7)	4(1.4)		
	About 6%	28(17.0)	35(12.6)		
	About 7%	45(27.3)	55(19.8)		
	About 8%	28(17.0)	57(20.5)		
	>9%	34(20.6)	119(42.8)		
Early DR presents with	Redness	8(4.8)	15(5.4)	6.538	0.088
	Pain	7(4.2)	29(10.4)		
	Blurry vision	95(57.6)	136(48.9)		
	Asymptomatic	55(33.3)	98(35.3)		

**Figure 5:**
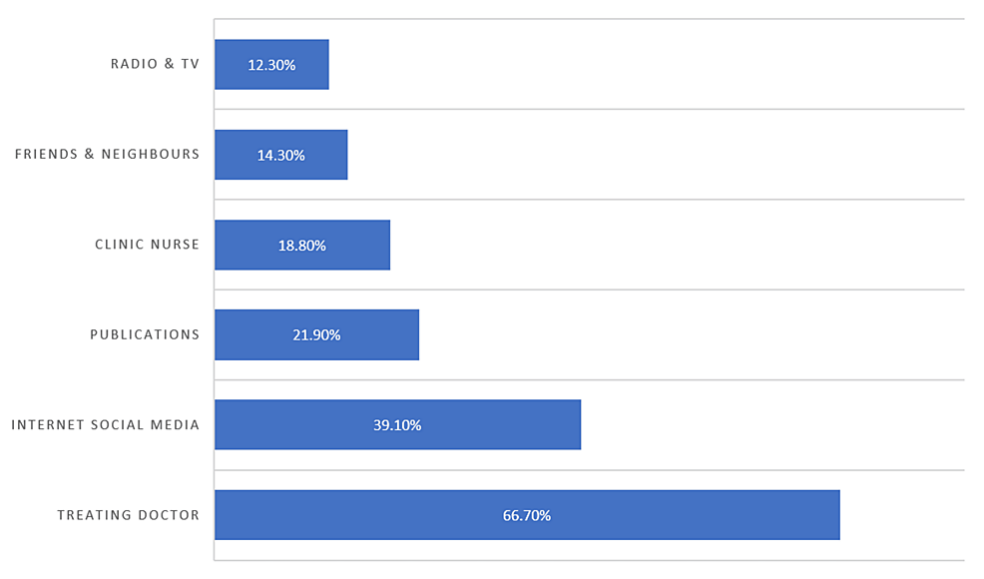
The main source(s) of information of diabetes mellitus and diabetic retinopathy. A responder can choose more than one option. The information is expressed in percentage of the total number of respondents.

The primary source of information about DM and DR for the majority of respondents was the treating physician, accounting for 299 (66.7%) of the participants. In contrast, television and radio were the least popular sources of information, reported by only 55 patients (12.3%) (Figure 5).

## Discussion

Globally, the prevalence of DM is increasing. According to WHO, about 422 million people worldwide have diabetes, with the majority living in low-and middle-income countries; diabetes is responsible for 1.5 million annual deaths [1]. The International Diabetes Federation identifies diabetes as one of the fastest-growing health emergencies of the twenty-first century [5]. The MENA region has the second highest DM prevalence worldwide, with 73 million adults (prevalence of 12.8%) estimated to be living with DM. This number is projected to reach 136 million by 2045, an increase of 96% [5]. Jordan is one of the MENA countries that is facing challenges with DM. In 2007, Prof. Ajlouni published research results reporting that the age-standardized prevalence of DM and IFG was 17.1% and 7.8%, respectively [6]. In 2019, the same researcher published that the prevalence of DM in men aged ≥ 25 years increased from 14.2% to 18.3% in 2004, to 26.8% in 2009, and to 32.4% in 2017 [7]. Out of Jordan’s total health expenditure, DM’s share was 19.0% in 1990 and is expected to increase to 25.2% by 2050 [8].

A major challenge for diabetics is the ocular complications of DM, specifically DR, which, along with cataracts, is considered the major cause of blindness in working-age adults. Awareness about DM and DR is crucial for combating DR, and many studies have been designed to measure this awareness. In Jordan, the awareness issue was examined for in different designs. Examples include a 2019 study surveying awareness about DM in public and not only in diabetics [9]. Another study published in 2022 tested diabetics in Amman (Capital) in tertiary and primary health centers [10]. Hence, we decided to test the awareness in diabetics being the targeted population for awareness, and the sample was intended to come from all sites of the country. We needed this study to plan a future screening and awareness program about DM and DR. The first phase [11] was a general survey to get a glimpse of the general trend and ideas about DM. In phase II, we needed to build a structured screening and awareness program to help Jordanian diabetics. The aim was to prioritize when and how to start and who to target most.

The first noticed challenge was to get each patient diagnosed with DM with regular follow-up by a treating doctor (internist, endocrinologist, general practitioner, or family doctor), with the awareness that more than a third (36.9%) are not followed up at all. Furthermore, more than a third had never been seen by an ophthalmologist (38.9%), and a similar percentage (34.2%) think that an optometrist visit is enough to detect DR. However, DR has many stages and normal vision is not a sign of exemption. A drop in vision can be a late sign of DR [12]. Although a large percentage (85.2%) are aware of the fact that DM affects the eye, almost two-thirds (60.9%) believe they need to have symptoms to seek an eye doctor checkup for DR. Compared to a similar study in SA, those aware of DM and DR connection are 79.5%; however, 48% believed they need to have eye symptoms to visit an eye doctor for DR checkup [13].

The second vital concern is poor BS control. In the mentioned study from SA, it was noted that more than half had poorly controlled DM [13], while in our study population, only 41.6% had controlled levels of HbA1c. Having said that, 68% know what HbA1c means, more than two-thirds get a regular BS checkup at home, and a similar percentage reported that they know DR is related to DR control. Although these facts have reached a large segment of the population, they are not reflected in BS control.

Referral to eye checks for DR should be routine for all diabetics upon diagnosis, particularly for DMT2, and DMT1 after the age of 12 [14]. It's not uncommon to find DR in newly diagnosed diabetic patients, especially considering that the diagnosis of DM can be delayed and missed for years [15,16]. In Jordan, DR screening is conducted by ophthalmologists. Emphasizing the need for an eye check upon DM diagnosis is crucial, particularly as this information is lacking across all education levels (school, college, and postgraduate).

Deciding which area to prioritize is crucial to extract the most benefit from the program, especially when starting with limited resources. The central area of Jordan is evidently more aware of DM and DR and probably has easier access to services. However, the southern and northern parts of the country lag behind in this regard.

The source of information for awareness varies across societies. In an Indian study, the treating doctor was the primary information source, with family members being the second source on the list [17]. Following the trend of the local community allows for utilizing the most trustworthy source of information. More investment in the social media route to spread information and awareness is advisable, noting that it ranks immediately after the “treating doctor” as the source of information for patients in our study population.

The main limitation of the study is collecting information via a link sent to the smartphones of patients or their companions. This approach likely misses a sector of patients who either do not have a smartphone, do not know how to fill out the form, or cannot read well due to vision limitations or literacy reasons.

## Conclusions

We concluded that awareness about DM and DR is not satisfactory to aid patients in their long journey with minimal, if any, complications. Enhancing awareness is crucial not only for better quality of life but also for maintaining economic and social balance in the community. The implementation of a sustainable program and a national awareness campaign utilizing social media is essential. To effectively manage the entire country, it is necessary to prioritize and begin with the north and south regions, before focusing on the middle regions of Jordan.
